# Risk prediction model for in-stent restenosis following PCI: a systematic review

**DOI:** 10.3389/fcvm.2024.1445076

**Published:** 2024-08-29

**Authors:** Qin Xiang, Xiao-Yun Xiong, Si Liu, Mei-Jun Zhang, Ying-Jie Li, Hui-Wen Wang, Rui Wu, Lu Chen

**Affiliations:** ^1^ Department of Nursing, The 2nd Affiliated Hospital, Jiangxi Medical College, Nanchang University, Nanchang, China; ^2^ School of Nursing, Jiangxi Medical College, Nanchang University, Nanchang, China

**Keywords:** coronary heart disease, PCI, restenosis, prediction model, systematic review

## Abstract

**Introduction:**

The morbidity and mortality rates of coronary heart disease are significant, with PCI being the primary treatment. The high incidence of ISR following PCI poses a challenge to its effectiveness. Currently, there are numerous studies on ISR risk prediction models after PCI, but the quality varies and there is still a lack of systematic evaluation and analysis.

**Methods:**

To systematically retrieve and evaluate the risk prediction models for ISR after PCI. A comprehensive search was conducted across 9 databases from inception to March 1, 2024. The screening of literature and extraction of data were independently carried out by two investigators, utilizing the checklist for critical appraisal and data extraction for systematic reviews of prediction modeling studies (CHARMS). Additionally, the risk of bias and applicability were evaluated using the Prediction Model Risk of Bias Assessment Tool (PROBAST).

**Results:**

A total of 17 studies with 29 models were included, with a sample size of 175–10,004 cases, and the incidence of outcome events was 5.79%–58.86%. The area under the receiver operating characteristic curve was 0.530–0.953. The top 5 predictors with high frequency were diabetes, number of diseased vessels, age, LDL-C and stent diameter. Bias risk assessment into the research of the risk of higher bias the applicability of the four study better.

**Discussion:**

The overall risk of bias in the current ISR risk prediction model post-PCI is deemed high. Moving forward, it is imperative to enhance study design and specify the reporting process, optimize and validate the model, and enhance its performance.

## 1 Introduction

Coronary heart disease (CHD) poses a significant threat to human life and health as a chronic disease, with its morbidity and mortality showing an increasing trend year by year (1). Percutaneous coronary intervention (PCI) is the primary treatment for CHD (2), offering the advantages of minimal trauma and fewer complications. In-stent restenosis (ISR) following PCI refers to the narrowing of the stent lumen or 5 mm segments at both ends of the stent detected by coronary angiography, resulting in a stenosis degree ≥50% (3). The main pathophysiological mechanism involves vascular endothelial injury and neointimal hyperplasia (4). ISR is a major cause of stent failure and repeated revascularization, serving as a key limitation for PCI treatment (5) and significantly impacting postoperative efficacy. Despite the reduction in ISR occurrence with drug-eluting stents (DES), its incidence remains high at 5%–10% (6). Furthermore, there is currently no standardized clinical treatment plan for ISR (7). Therefore, risk stratification based on risk factors plays a crucial role in identifying high-risk populations for ISR after PCI early on and implementing timely intervention measures. Clinical prediction model (8), as an assessment tool, can evaluate the current health status of patients, help medical staff quantify the risk value of a patient's future disease, and also provide individualized medical evidence for patients, which is beneficial to improve medical cost efficiency. It may even affect the diagnosis and outcome (9). Combining multiple variables or features to construct a prediction model for ISR is helpful to understand the pathogenic mechanism of the combined effect of multiple risk factors. This method helps to have more accurate on the onset and progress of ISR evidence-based knowledge, also is advantageous to the medical staff to make the most benefit at the patient's clinical decision, to optimize clinical outcomes (10). Researchers both domestically and internationally have conducted studies on ISR risk factors after PCI, continuously developing or validating various risk prediction models; however, these models vary in research quality. Thus, this study aims to analyze and compare the fundamental characteristics, modeling methods, predictive variables, and methodological quality of ISR risk prediction models for patients after PCI to offer guidance for clinical medical staff when selecting or developing appropriate ISR risk prediction models after PCI.

## 2 Methods

In this study, the PICOTS model, as suggested by the Cochrane Prognostic Methods Group, was employed to formulate evidence-based inquiries (11). The target population (P) comprised patients experiencing restenosis after PCI. The index prediction model (I) under investigation was the ISR risk prediction model. There was no specific comparator (C) identified. The primary outcome (O) of interest was the occurrence of stent restenosis post-PCI. The model's application timeframe (T) was during angiography following PCI. The setting (S) for utilizing the model was within the domain of cardiovascular medicine. Apart from that, we use the Checklist for Critical Appraisal and Data Extraction for Systematic Reviews of Risk Prediction Models (CHARMS) by Moons et al. (12) was employed for screening literature and extracting data. Furthermore, the Prediction Model Risk of Bias Assessment Tool (PROBAST) developed by Moons et al. (13) was utilized to evaluate the risk of bias and the applicability of quality assessment for research on ISR prediction models.

### 2.1 Retrieval strategy

We conducted a thorough computerized search across multiple databases, including PubMed, Web of Science, Embase, Cochrane Library, CINAHL, Chinese Biomedical Literature Database (CBM), China National Knowledge Infrastructure (CNKI), Wanfang, and Weipu (VIP). The search spanned from the inception of each respective database up to March 1, 2024.

The search strategy is as follows: using medical subject headings (MeSH), subject headings, abstract and keyword combinations, Key words include: “Percutaneous Coronary Intervention/Coronary Intervention, Percutaneous/Intervention,Percutaneous Coronary/Coronary Revascularization,Percutaneous/PCI""Coronary restenosis/Restenosis, Coronary/restenosis/restenosis lesions/instent restenosis""risk assessment/predict*/prediction model/risk prediction/risk factors”. In addition, we manually searched the references of the included studies. Taking PubMed as an example, the search strategy is as follows: (((((Percutaneous Coronary Intervention[MeSH Terms]) OR (Percutaneous Coronary Intervention[Title/Abstract])) OR (Coronary Intervention*,Percutaneous[Title/Abstract])) OR (Intervention,Percutaneous Coronary*[Title/Abstract])) OR (Coronary Revascularization,Percutaneous[Title/Abstract])) OR (PCI[Title/Abstract]))))) AND ((((((Coronary restenosis[MeSH Terms]) OR (Restenosis*, Coronary[MeSH Terms])) OR (Coronary restenosis[Title/Abstract])) OR (Restenosis*, Coronary[Title/Abstract])) OR (restenosis[Title/Abstract])) OR (restenosis lesions[Title/Abstract])) OR (instent restenosis[Title/Abstract])))))) AND (((((risk assessment[MeSH Terms]) OR (risk assessment[Title/Abstract])) OR (predict*[Title/Abstract])) OR (prediction model[Title/Abstract])) OR (risk prediction[Title/Abstract])) OR (risk factors*[Title/Abstract])))))).

### 2.2 Study screening and inclusion and exclusion criteria

**Figure 1:**
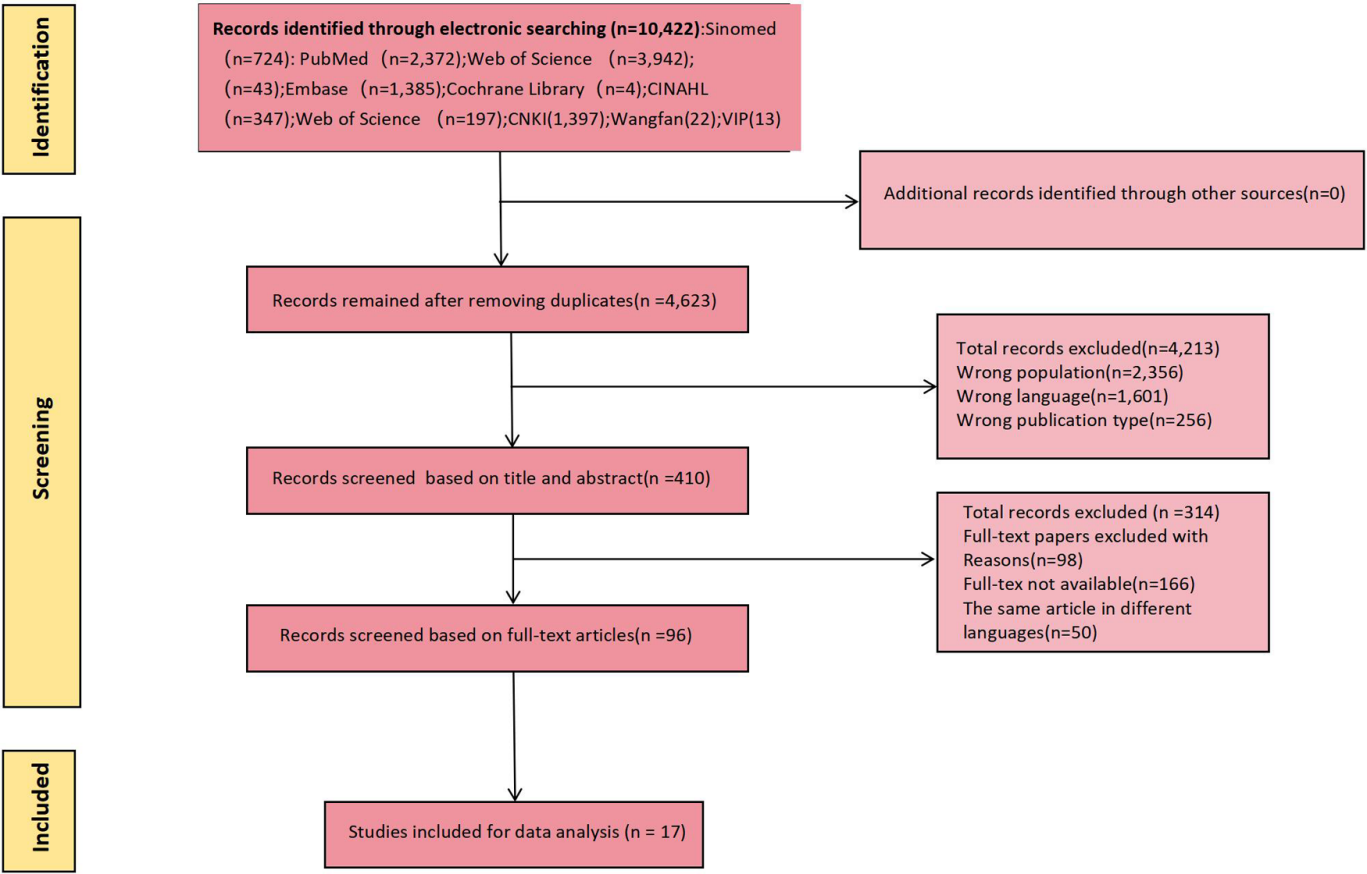
Flow chart of literature screening.

Use the Endnote software delete found in the database to retrieve all repeated study, two researchers independent screening the rest of the title and abstract, the full text of selected independent review to ensure that the report up to the standard. Study inclusion criteria were as follows: (1) Patients aged ≥18 years old with ISR after PCI; (2) The research content was the construction and/or validation of ISR risk prediction model after PCI; (3) Study types were cross-sectional study, case-control study or cohort study; (4) Chinese and English studies. Exclusion criteria: (1) The model included less than 2 predictors; (2) Unable to extract complete data; (3) unable to get the full text; (4) Repeated publication; (5) The specific process of modeling was not described. Study screening and data extraction were cross-checked by two researchers independently, and a third party was consulted in case of disagreement. See Figure 1 for detail.

### 2.3 Data extraction

Data from the included studies were independently extracted by two investigators and subsequently cross-checked by a third investigator. Any discrepancies or inconsistencies were resolved through discussion among the investigators or by seeking professional consultation. Utilizing a standardized data collection form, CHARMS data were extracted from the included studies. The form comprises the following details: Year of publication, country of study, study design type, research object, study site, and outcome definition. Additionally, it includes information on the number of candidate variables, continuous variable processing method, sample size, proportion of restenosis events and other information.

### 2.4 Risk of bias in the studies

Two researchers independently utilized the Prediction Model Risk of Bias Assessment Tool (PROBAST) (13) to assess the risk of bias and applicability of the included studies. In instances of disagreement, a third party was consulted for resolution. The risk of bias assessment encompassed four aspects: subjects, predictors, outcomes, and data analysis, with a total of 20 questions. Applicability was evaluated across three dimensions: subjects, predictors, and outcomes. Each question could answer “Yes (Y)/Probably Yes (PY),” “Probably Not (PN)/No (N),” or “No Information (NI).” Each domain and overall will be assessed as “high risk of bias,” “low risk of bias,” or “unclear risk of bias.” Applicability is rated as “good applicability,” “poor applicability,” or “unclear applicability.” When the evaluation results of all domains are low risk of bias or good applicability, the overall risk of bias and applicability are judged to be low risk of bias or good applicability.

### 2.5 Statistical analysis

Throughout the study, descriptive statistics and narrative reviews were utilized for organizing and synthesizing research findings (14), while thematic analysis (15) was employed for categorizing influencing factors.

## 3 Results

### 3.1 Data search and included literature

A total of 10,422 relevant studies were initially retrieved, from which 17 were ultimately included following screening (16–32). This comprised 13 studies in English and 4 studies in Chinese. The literature screening process is illustrated in Figure 1.

### 3.2 Study characteristics

Studies were published between 2013 and 2024, with the majority originating from China (*n* = 12), followed by Germany (*n* = 2), and one each from Romania, Spain, and South Korea. Among these, three (16, 18, 21) were prospective studies, while the remaining were retrospective. A total of 29 models were included, with sample sizes ranging from 175 to 10,004, and the incidence of outcome events varying from 5.79% to 58.86%. Logistic regression methods were employed in 11 studies (16–21, 23, 25, 27–30, 32) see Table 1.

**Table 1:** Basic characteristics of the included studies (*n* = 17).

Study	Publication time (year)	Country/Region	Study type	Research object	Data source location	Outcome definition
Qian et al.	2024	China	Prospective cohort study	CHD	Hai’an area, Jiangsu Province	Coronary angiography or coronary CT angiography showed that the stent was located in the coronary artery, and the degree of lumen stenosis ≥50%
Bai et al.	2024	China	Case control study	AMI	Guizhou Province	If AMI recurs within 28 days, restenosis may occur in the original site or other myocardial sites
Wu et al.	2023	China	Prospective cohort study	CHD	Shandong Provincial Third Hospital Affiliated to Shandong University	Lumen stenosis of ≥50% in the stent-implanted segment at 12-month follow-up compared with lumen assessed immediately after PCI
Jia et al.	2023	China	Case control study	Hypertension and CHD	Fuyang Second People's Hospital	Confirmed by coronary angiography and stent implantation stent, 5 mm of the distal segment lumen diameter stenosis 50% or higher
Scafa-udriste et al.	2023	Romania	Retrospective case control study	ACS	Bucharest clinical acute care hospitals	Lumen narrowing accept percutaneous coronary interention (PCI), myocardial contraction of more than 50%, or at least 5 mm on the edge of the bracket
Guldener et al.	2023	Germany	Prospective cohort study	CHD	Two centers in Munich	Coronary angiography showed intrasegmental diameter stenosis ≥50%
Coughlan et al.	2023	Germany	Retrospective study	Myocardial infarction	Two centers in Germany	Any repeat percutaneous intervention was performed on the restenosis lesion targeted by the initial treatment
Jiang et al.	2022	China	Retrospective cohort study	CHD	Guizhou Provincial People's Hospital	Stenosis of more than 50% at 5 mm within or adjacent to the segment in which the stent was previously placed was analyzed by quantitative coronary artery
Study	Publication time (year)	Country/Region	Research type	Research object	Data source location	Outcome definition
Luo et al.	2022	China	Retrospective study	CHD	Enshi Central Hospital	Angiographic lumen stenosis ≥50%
Lin et al.	2022	China	Retrospective cohort study	ACS	Beijing Anzhen Hospital Affiliated to Capital Medical University	The restenosis of the arterial lumen within 5 mm of the stent segment or the proximal or distal end of the stent area after PCI was ≥50%
Yi et al.	2022	China	Retrospective cohort study	CHD with Diabetes mellitus	Zhongshan Cardiovascular Hospital, Shenzhen	Support period of luminal stenosis vascular angiography confirmed 50% or higher
Wang et al.	2022	China	Retrospective case control study	CHD	The First Affiliated Hospital, Xinjiang Medical University	Newly found plaque in the stent or within 5 mm from the edge of the stent after PCI, and the degree of stenosis ≥50% was defined as vasoproliferative disease
He et al.	2021	China	Retrospective study	CHD	Henan province	Angiographic lumen stenosis ≥50%
Gai et al.	2021	China	Retrospective cohort study	CHD	Xinjiang	The degree of coronary angiographic restenosis was more than 50%, including the original stent placement site and the vascular segment 5 mm away from the stent
Zhao et al.	2020	China	Retrospective study	CHD	The Second Hospital of Hebei Medical University	The percentage diameter stenosis in the stent-implanted segment at the 1-year follow-up was >50% as compared with the lumen assessed immediately after PCI
Jesús et al.	2020	Spain	Retrospective study	CHD	20 Spanish hospitals	All underwent partial blood vessel scaffold area treatment including the edge of the proximal and distal 5 mm vascular lumen diameter of >50%
Kang et al.	2013	Korea	Retrospective study	CHD	Seoul	The diameter stenosis was >50% at angiography

CHD, coronary heart disease; ACS, acute coronary syndrome; AMI, acute myocardial infarction.

### 3.3 Model development and performance

Among the 29 included models, 27 (16–21, 23–25, 27–32) reported areas under the receiver operating characteristic curve (AUC) ranging from 0.460 to 0.953. Except for the 7 models constructed by Scafa-udriste et al. (20), Lin et al. (25), He et al. (28), and Kang et al. (32), the AUC values of the other models exceeded 0.7. The calibration of five models 27 (17, 24, 28, 29) was evaluated using the Hosmer-Lemeshow test. Three studies (16, 28, 31) reported missing data, with two studies (28, 31) employing specific treatment methods such as direct deletion and data imputation, respectively, while six studies (17, 18, 20, 27, 30, 32) did not report the presence or absence of missing values. Table 2 shows the performance and predictors of all models.

**Table 2:** Establishment, validation, variables and model performance of ISR risk prediction model for patients after PCI (*n* = 29).

First author	Number of candidate variables (n)	Continuous variable processing method	Sample size [Modeling/validation (example)]	Proportion of restenosis events (%)	Number of missing data and processing method	Modeling method	Model AUC	Method of calibration	Model validation methods
Qian et al.	25	Partial conversion to classification	1,689	7.62	153,-	Logistic regression	0.843 (0.813–0.887)	Curve of calibration	1,000 bootstrap sampling method
Bai et al.	9	Partial conversion to classification	1,359/395	13.61/8.61	–	Logistic regression	A:0.800 (0.770–0.840); B:0.760 (0.710–0.810)	H-L goodness of Fit	Internal validation
Wu et al.	24	Continuity	320	11.25	–	Random forest algorithm	0.916	Curve of calibration	Bootstrap method
Jia et al.	20	Partial conversion to classification	182	23.08	0,-	Logistic regression	0.834 (0.806–0.862)	Curve of calibration	–
Scafa-udriste et al.	21	Continuity	340	17.68	–	The depth of the neural network; linear logistic regression; Random forest; Support vector machine	A:0.695; B:0.660; C:0.694; D:0.721 (0.709–0.733)	–	Randomly split validation
Guldener et al.	33	Continuity	10,004	56.90	0,-	Regressive neural network	A:0.728; B:0.726	Curve of calibration	Randomly split validation
Coughlan et al.	35	Continuity	1,471/515	16.80	0,-	LASSON regression	–	–	Randomly split validation
Jiang et al.	15	Continuity	1,501	18.60	0,-	Logistic regression	A:0.829 ± 0.025; B:0.784 ± 0.027	Curve of calibration	Randomly split validation
Luo et al.	35	Continuity	477	19.70	0,-	LASSON regression	0.841	H-L goodness of fit	1,000 bootstrap sampling method
Lin et al.	49	Continuity	797	25.40	0,-	Logistic regression	A:0.714; B:0.692	–	2,000 bootstrap sampling method
Yi et al.	30	Continuity	1,741	18.60	0,-	COX proportional risk model	–	Curve of calibration	Internal validation
Wang et al.	24	Partial conversion to classification	472	26.67; 23.62	–	Logistic regression	A:0.905; B:0.807	Curve of calibration	–
He et al.	36	Partial conversion to classification	325/138	23.90	116, direct deletion	Logistic regression	0.662	H-L goodness of fit	Randomly split validation
Gai et al.	42	Continuity	968	5.79	0,-	Logistic regression	0.720 (0.640–0.800)	H-L goodness of Fit;Curve of calibration	1,000 bootstrap sampling method
Zhao et al.	42	Continuity	398	9.30	–	Logistic regression	0.953 (0.926–0.981)	Curve of calibration	–
Jesús et al.	68	–	299	9.00	36, Data filling	Random forest; Extreme random tree; Gradient rise; Support vector machine classifier; Logistic regression	A:0.750 (0.630–0.880); B:0.770 (0.660–0.890); C:0.670 (0.520–0.830); D:0.530 (0.370–0.680); E:0.770 (0.650–0.880)	Curve of calibration	K-fold cross validation; external validation
Kang et al.	40	Continuity	290	58.86	–	Logistic regression	0.756	Curve of calibration	–

– : no description.

### 3.4 Predictive variables included in the prediction model for ISR after PCI

The number of predictors included in the models ranged from 3 to 19. According to the thematic analysis method, the risk factors involved in the included studies were classified and described, which could be divided into three categories: disease and treatment factors, laboratory indicators, and demographic characteristics. The top 5 predictive variables consistently reported by the models were diabetes mellitus (*n* = 8), number of diseased vessels (*n* = 6), age (*n* = 6), LDL-C (*n* = 5), and stent diameter (*n* = 5). see Tables 3, 4 for more details.

**Table 3:** Risk predictors of ISR after PCI.

Author	Predictor variable
Qian et al.	5: diabetes mellitus, number of diseased vessels, low-density lipoprotein cholesterol, number of implanted stents, and minimum stent diameter
Bai et al.	4: family history of coronary heart disease and diabetes mellitus, high-sensitivity troponin I >0.342 μg/L, LDL-C ≥3.37 mmol/L, and lack of exercise after surgery
Wu et al.	7: diabetes, hyperlipidemia, age, three acyl glycerin and high-density lipoprotein cholesterol ratio, glycosylated hemoglobin, three acyl glycerin—glycemic index, hypersensitive c-reactive protein
Jia et al.	7: diabetes, chronic cigarette smoking, high uric acid, high-sensitivity c-reactive protein (>10 g/L), serum amyloid A (10 mg/L or higher), lipoprotein (A) 300 mg/L, or stent diameter 3 mm or less
Scafa-udriste et al.	3: the number of affected arteries (≥2), stent type, and stent diameter
Guldener et al.	18: diabetes mellitus, hypercholesterolemia, chronic vascular occlusion, history of PCI, history of bypass surgery, complex lesion morphology, severity of stenosis, reduction in vessel size, and clinical presentation (variables ranked by severity: St-segment elevation myocardial infarction, non-ST-segment elevation acute coronary syndrome, stable angina), lesion length, final diameter stenosis achieved by PCI, age, BMI, thrombolysis in myocardial infarction flow in treated coronary vessels before PCI, stent type, stent length, balloon to vessel ratio, and left main stent
Coughlan et al.	4: left circumflex artery ISR, calcified vessels, non-focal ISR pattern, ISR interval <6 months
Jiang et al.	11: diabetes, hypertension, number of stenosis vessels, complex lesions (type B2 and C), age, male, smoking history, dyslipidemia, total stent length, minimum stent diameter, and ACS stent placement procedure
Luo et al.	5: chronic obstructive pulmonary disease, remnant cholesterol, neutrophils/rate of lymphocytes, monocytes, Gensini score high
Lin et al.	15: a history of diabetes, hypertension, myocardial infarction, PCI, single or multiple/left main disease, age, BMI, fasting glucose, glycosylated hemoglobin, low density lipoprotein cholesterol, left ventricular ejection fraction, glycated albumin, support number (2) or higher, the total length of scaffolds, minimum length diameter
Yi et al.	6: multivessel coronary artery disease, coronary artery diffuse lesions, and glomerular filtration rate <60 ml/min/1.73 m^2^, fasting blood glucose or greater tendency for 6.5/L, PCI operation time (60 min) or higher, emergency PCI
Wang et al.	6: history of diabetes, apolipoprotein B (ApoB), Gensini score, age, triglyceride, total cholesterol
He et al.	5: history of PCI, high blood glucose, stent type, lack of clopidogrel, and left anterior descending stent
Gai et al.	5: diseased vessels, platelet distribution width, total cholesterol, systolic blood pressure, and low-density lipoprotein cholesterol
Zhao et al.	6: hypertension, diabetes mellitus, left circumflex artery target lesion, age, low-density lipoprotein cholesterol, high-sensitivity C-reactive protein
Jesús et al.	6: diabetes mellitus, ≥2 coronary artery disease, thrombolytic therapy for myocardial infarction after PCI, thrombosis after PCI, platelet, cholesterol
Kang et al.	6: diabetes mellitus, angiographic segmental diameter stenosis, IVUS minimum lumen area, proximal location of IVUS minimum lumen area, multifocal or diffuse in-stent ISR pattern, insufficient stent expansion (minimum stent area <5.0 mm^2^)

**Table 4:** Categories of risk predictors for ISR after PCI.

Theme	Risk factors
Disease and treatment factors	Previous PCI history, number of diseased vessels, blood flow velocity, number and size of stents deployed, stent type, procedure duration, presence of diabetes mellitus, hypertension, COPD status, and Gensini score
Laboratory indicators	Low-density lipoprotein cholesterol (LDL-C), high-sensitivity troponin, triglyceride to high-density lipoprotein cholesterol ratio (TG/HDL-C), glycosylated hemoglobin A1c (HbA1c), triacylglycerol-glucose index (TyG), high-sensitivity C-reactive protein (hs-CRP), uric acid, serum amyloid A (SAA), lipoprotein (a), Thrombolysis in Myocardial Infarction flow (TIMI), neutrophil-to-lymphocyte ratio, and monocyte count
Demographic characteristics	Age, family history, smoking status, BMI, male gender

### 3.5 Quality assessment

Among the 17 included studies, 15 (16–25, 27, 28, 30–32) were deemed to have a high risk of bias, while 2 (26, 29) could not be definitively assessed for bias. Concerning study subjects, nine studies (16, 20–25, 28, 32) were rated as high risk, seven studies (17–19, 26, 27, 29, 30) as low risk, and one study (31) as unclear risk. Regarding predictors, four studies (18–20, 25) were judged to have a high risk of bias, ten studies (17, 21, 22, 26–32) as low risk, and three studies (16, 23, 24) as unclear. Concerning outcomes, seven studies (16, 18, 19, 25, 30–32) were deemed to have a high risk of bias, four studies (24, 26, 27, 29) as unclear, and the remainder as low risk. For data analysis, five studies (17, 27, 28, 31) were rated as high risk, six studies (16, 18, 19, 21, 22, 29, 32) had unclear risk, and the rest were deemed low risk. Four studies (21, 23, 24, 30) received favorable applicability evaluations across study subjects, predictors, and outcomes, indicating high overall applicability, while the remaining studies scored low in applicability assessments. See Table 5 and Supplementary Appendix 1.

**Table 5:** Included in the study of risk of bias and applicability evaluation (*n* = 17).

First author	Deviation risk				Applicability			Overall applicability	
	Subjects	Predictors	Outcome	Data Analysis	Subjects	Predictors	Outcome	Risk of bias	Applicability
Qian et al.	H	U	H	H	L	H	H	H	L
Bai et al.	L	L	L	H	H	L	H	H	L
Wu et al.	L	H	H	U	H	H	L	H	L
Jia et al.	L	H	H	U	L	H	H	H	L
Scafa-udriste et al.	H	H	L	L	H	L	H	H	L
Guldener et al.	H	L	L	U	H	H	H	H	H
Coughlan et al.	H	L	L	U	H	L	H	H	L
Jiang et al.	H	U	L	L	H	H	H	H	H
Luo et al.	H	U	U	L	H	H	H	H	H
Lin et al.	H	H	H	L	L	H	H	H	L
Yi et al.	L	L	U	L	H	L	L	U	L
Wang et al.	L	L	U	H	H	L	H	H	L
He et al.	H	L	L	H	L	H	H	H	L
Gai et al.	L	L	U	U	L	H	H	U	L
Zhao et al.	L	L	H	L	H	H	H	H	H
Jesús et al.	U	L	H	H	L	H	H	H	L
Kang et al.	H	L	H	U	L	H	L	H	L

H, high.

L, low.

U, unclear.

## 4 Discussion

### 4.1 Summary of main findings

We conducted a systematic review focusing on the application of clinical prediction models in studies concerning the diagnosis and prognosis prediction of in-stent restenosis (ISR) following percutaneous coronary intervention (PCI). Upon screening the 17 finally included studies, we found that the area under the receiver operating characteristic curve (AUC) of ISR prediction models after PCI ranged from 0.530 to 0.953. With the exception of four models (20, 25, 28, 31) with AUC values below 0.70, the remaining models exhibited satisfactory prediction performance (AUC > 0.70). However, it's worth noting that all 17 studies included in this review demonstrated a high risk of bias. This indicates that there's a need for future studies to enhance their study design and reporting processes, optimize and validate the models, and ultimately improve their performance.

### 4.2 High bias risk analysis of ISR risk prediction model after PCI

All the 17 studies included in this study showed a high risk of bias. The main reasons for this phenomenon are as follows: (1) Inadequate data size: Ten studies (18–20, 24, 25, 27, 28, 30–32) suffered from small sample sizes (sample size/candidate variables < 20), potentially predisposing the models to overfitting. (2) Inadequate handling of missing data: Among the studies included, six (17, 18, 20, 27, 30, 32) did not report any missing data, three (16, 28, 31) specified the presence of missing data, and one (16) did not detail the specific handling methods for missing data. Proper handling of missing data is crucial for reducing bias. Currently, various methods exist for addressing missing data, such as weighting methods, among others. Employing appropriate techniques tailored to the specific circumstances can mitigate the adverse effects of missing data on statistical analysis and model reliability. (3) Improvement needed in validation methods: While five studies (16, 18, 24, 25, 29) utilized the Bootstrap self-sampling method, the majority of included studies relied on internal validation methods, primarily random split validation. However, this validation approach not only diminishes the effective sample size for model development but also heightens the risk of overfitting. (4) Reliance on a single data analysis method: Eleven studies (16, 17, 19, 20, 23, 25, 27–30, 32) employed logistic regression analysis to construct models. However, contemporary approaches encompass various data analysis methods, including deep learning and decision trees, for handling complex data. Hence, alongside traditional prediction methods, integrating alternative techniques can enhance the robustness of prediction models. The initial step in prevention involves conducting risk assessment and prediction. The accuracy of predictive results will directly impact the selection and effectiveness of preventive management measures. Bias is usually defined as the presence of a systematic error that may affect the study's validity (33). A high overall bias in risk prediction models may lead to the following issues: inaccurate predictive results; suboptimal decision-making; bias in resource allocation; trust issues. Suggestions for future research involve augmenting sample sizes, enhancing the standardization of data reporting and processing, refining validation methods, and incorporating diverse data analysis methodologies. Adhering to standardized research methods and reporting processes can bolster the quality of investigations, furnishing a more scientific and reliable foundation for clinical decision-making.

### 4.3 High risk factors of ISR risk prediction model after PCI

Among the 17 predictors finally included in the study, spanning from 3 to 18, they can be categorized into three types. Analysis revealed that among the 22 models considered, the most prevalent predictors were diabetes mellitus, the number of diseased vessels, age, LDL-C, and stent diameter.

Diabetes is widely recognized as a risk factor for intrastent restenosis after PCI (34), and was also noted in 10 studies (16, 18, 19, 21, 23, 25, 27, 30–32) in this study. This association can be attributed to several factors. Firstly, in individuals with diabetes, chronically elevated blood glucose levels can directly or indirectly stimulate the generation of reactive oxygen species, inflammation, and metabolic factors within the body. These physiological processes can adversely affect the repair of blood vessels where stents are placed. Secondly, insulin resistance in patients with type II diabetes mellitus can easily lead to lipid deposition in the intima of blood vessels, trigger atherosclerosis and inflammatory reaction of the vessel wall, thereby stimulating the inflammatory reaction of the vessel wall, promoting the formation of thrombosis and accelerating the occurrence of ISR. In patients with abnormal lipid metabolism, lipids are more likely to be deposited in the vessel wall, thereby increasing the risk of ISR (35, 36).

The risk of ISR is high in elderly patients. Due to the relatively high number of underlying diseases, the compensatory capacity of various organs is decreased, and the repair of vascular endothelial function is impaired. In addition, the arterial wall of elderly patients is relatively thickened, the anticoagulant ability is weakened, and they are more likely to suffer from atherosclerosis (37).

Elevated levels of LDL-C can disrupt the structure and function of vascular endothelial cells, trigger inflammation, while reducing LDL-C concentration can delay the onset of ISR (34).

Stent implantation itself can cause vascular damage, and with the more and longer stents inserted, the greater vascular resistance and the greater pressure required for stent release will further damage vascular endothelial cells, leading to platelet adhesion and intimal hyperplasia, leading to ISR (34). In addition, bare metal stents can greatly increase the risk of ISR, and drug-eluting stents can inhibit endothelial cell proliferation for a long time (34).

Surgical history is a widely accepted risk factor for in-stent restenosis after PCI (38, 39). Surgery can cause different degrees of damage to the vascular wall, which leads to endothelial cell stripping, inflammatory reaction, intimal repair proliferation and smooth muscle cell proliferation. The more the number of operations, the greater the damage to the vascular wall, and inflammation and repair lead to stent restenosis.

In addition, patients with smaller vessels have a high risk of restenosis due to the fact that they are more likely to develop diseases such as diabetes or multivessel coronary artery disease, resulting from multiple factors (40).

Despite the increasing number of studies on in-stent restenosis after PCI, recognized risk factors include: the type of implanted stent, the presence of diabetes mellitus, previous bypass surgery, and small vessel caliber. However, the exact cause of in-stent restenosis after PCI is still controversial, and the risk factors are complex and diverse. Therefore, it is particularly important to carry out differential risk stratification (39) and accurately identify the predictors related to patients, stents and lesions, which is also the deficiency of existing studies.

Hence, in clinical practice, it is imperative to conduct a comprehensive assessment of individual risk factors, closely monitor these high-risk patients, and devise personalized multidisciplinary intervention programs to preempt the occurrence and progression of ISR.

### 4.4 Application of prediction models in clinical practice

The risk score within the prediction model relies on readily accessible clinical and laboratory data, facilitating easy calculation and implementation (41). This offers a swift and convenient means for clinics to quantify risk, aiding in the formulation of individualized patient treatments. In clinical practice, prediction models serve to unveil the risk of ISR post-PCI, and accurate models empower medical professionals to make optimal clinical decisions or guide adjunctive therapies. Hence, it is essential for them to grasp the strengths and limitations of these models.

Currently, research on ISR risk prediction models post-PCI is progressively advancing. Our study underscores that while the included ISR risk prediction models post-PCI demonstrate partial applicability, they exhibit an overall high risk of bias. This underscores the imperative for further investigations in the future, such as augmenting sample sizes and extending follow-up periods. Such endeavors are pivotal for refining the accuracy and reliability of these predictive models.

There are a variety of machine learning algorithms that can be used to establish risk prediction models in computer and medical fields, and it has been confirmed that models established by machine algorithms have better prediction performance (42, 43). Therefore, in the future, modeling methods should be used reasonably to improve model performance. To facilitate clinical practice.

Furthermore, the absence of external validation in some studies hampers the universality of prediction models. Limited clinical research exists on whether these prediction models for ISR post-PCI are conducive to early identification and intervention. These shortcomings impede the refinement of prediction models in the post-PCI population and in clinical practice. Future research endeavors should focus on enhancing the clinical utility and simplifying the prediction model for ISR post-PCI, such as refining risk calculation formulas and graphical representations.

Establishing a comprehensive clinical medical decision system through prediction models can assist clinicians in diagnosing and devising personalized treatment plans for post-PCI patients. Based on our study, we recommend that healthcare practitioners adhere strictly to PROBAST report specifications when constructing future models. Additionally, there is a need to bolster multicenter, large-sample external validation efforts and integrate clinical insights to further validate the feasibility and applicability of these models in clinical settings. Moreover, continual updates, optimization, and calibration of the model's predictive power are imperative to furnish a robust foundation for high-quality clinical decision-making.

## 5 Strengths and limitations

This study has several significant advantages. Firstly, it provides a comprehensive review encompassing all relevant studies on ISR prediction models post-PCI, ensuring a thorough examination of existing literature. Secondly, by employing the PROBAST tool, we meticulously assess model bias and applicability, thereby providing a robust framework for evaluating various diagnostic or prognostic prediction models.

However, we must also acknowledge some limitations. While our primary focus is on evaluating the predictive performance and bias of the included models, we did not directly assess the applicability of the clinical prediction tool in real-world clinical settings. Additionally, the formulation of inclusion and exclusion criteria, along with subjective judgments made by investigators, may lead to omissions in the reviewed literature. Finally, due to language barriers, our study is limited to published Chinese or English literature, which may introduce a certain degree of selection bias.

## 6 Conclusions

A total of 17 studies, encompassing 29 prediction models, were included in the analysis. The findings suggest that the current prediction models for in-stent restenosis (ISR) following PCI exhibit a high risk of bias overall, with only a minority of the models undergoing external validation. Hence, it is recommended that future research strictly adhere to the reporting steps outlined in PROBAST during model construction. Furthermore, there is a need to strengthen external validation through multi-center studies with large sample sizes. It is essential to further assess the applicability and feasibility of these models in clinical practice, considering the actual clinical context. By doing so, we can furnish a high-quality foundation for clinical decision-making.

## Data Availability

The original contributions presented in the study are included in the article/Supplementary Material, further inquiries can be directed to the corresponding author.

## References

[1] Myers J Fonda H Vasanawala M Chung K Segall G Chan K PCI alternative using sustained exercise (PAUSE): rationale and trial design. Contemp Clin Trials. (2019) 79:37–43. 10.1016/j.cct.2019.02.010 30797041

[2] Demyanets S Tentzeris I Jarai R Katsaros K-M Farhan S Wonnerth A An increase of interleukin-33 serum levels after coronary stent implantation is associated with coronary in-stent restenosis. Cytokine. (2014) 2:65–70. 10.1016/j.cyto.2014.02.014 24725541 PMC3996548

[3] Zhang Q Li T Chen R Zeng Z-H. Research progress of SGLT2i in the prevention of in-stent restenosis after percutaneous coronary intervention. J Pract Med. (2024) 40(08):1175–80. 10.3969/j.issn.1006-5725.2024.08.027

[4] Yang S-M Li S-C Geng C-Q Gao F. Research progress of in-stent restenosis after percutaneous coronary intervention. Combine Trad Chin West Med Jo Cardio Cerebrovasc Dis. (2023) 21(20):3754–60. 10.12102/j.issn.1672-1349.2023.20.013

[5] Wang F Li C Ding F-H Shen Y Gao J Liu Z-H Increased serum TREM-1 level is associated with in-stent restenosis, and activation of TREM-1 promotes inflammation, proliferation and migration in vascular smooth muscle cells. Atherosclerosis. (2017):26710–18. 10.1016/j.atherosclerosis.2017.10.015 29080545

[6] Erdogan E Bajaj R Lansky A Mathur A Baumbach A Bourantas C-V. Intravascular imaging for guiding in-stent restenosis and stent thrombosis therapy. J Am Heart Assoc. (2022) 11(22):e26492. 10.1161/JAHA.122.026492 36326067 PMC9750080

[7] Ma W-L Liu Q-F Bao F-M Cai Y Zhang F-M. The application value of NLR, MHR and their combination in the diagnosis of in-stent restenosis after percutaneous coronary intervention in patients with coronary heart disease. Guangxi Med. (2021) 43(8):913–6. 10.11675/j.issn.0253-4304.2021.08.02

[8] Meehan A-J Lewis S-J Fazel S Fusar-Poli P Steyerberg E-W Stahl D Clinical prediction models in psychiatry: a systematic review of two decades of progress and challenges. Mol Psychiatry. (2022) 27(6):2700–8. 10.1038/s41380-022-01528-4 35365801 PMC9156409

[9] Mvs A Jbr A Rdr B Gscc D Kgm A. Clinical prediction models: diagnosis versus prognosis. J Clin Epidemiol. (2021) 132:142–5. 10.1016/j.jclinepi.2021.01.009 33775387

[10] Collins G-S Reitsma J-B Altman D-G Moons K-G. Transparent reporting of a multivariable prediction model for individual prognosis or diagnosis (TRIPOD): the TRIPOD statement. Br Med J. (2015) 350:7594. 10.1136/bmj.g7594 25569120

[11] Moons K-G Hooft L Williams K Hayden J-A Damen J-A-A-G Riley R-D. Implementing systematic reviews of prognosis studies in Cochrane. Cochrane Database Syst Rev. (2018) 10(10):ED129. 10.1002/14651858 PMC10284248 30306538

[12] Moons K-G De Groot J-A Bouwmeester W Vergouwe Y Mallett S Altman D-G Critical appraisal and data extraction for systematic reviews of prediction modelling studies: the CHARMS checklist. PLoS Med. (2014) 11(10):e1001744. 10.1371/journal.pmed.1001744 25314315 PMC4196729

[13] Moons K-G-M Wolff R-F Riley R-D Whiting P-F Westwood M Collins G-S PROBAST: a tool to assess risk of bias and applicability of prediction model studies: explanation and elaboration. Ann Intern Med. (2019) 170(1):W1–33. 10.7326/M18-1377 30596876

[14] Stang A. Critical evaluation of the Newcastle-Ottawa scale for the assessment of the quality of nonrandomized studies in meta-analyses. Eur J Epidemiol. (2010) 25(9):603–5. 10.1007/s10654-010-9491-z 20652370

[15] Braun V Clarke V. Using thematic analysis in psychology. Qual Res Psychol. (2006) 3(2):77–101. 10.1191/1478088706qp063oa

[16] Qian J Gu S-Z Yan Y-J Lu Y. Prevalence of in-stent restenosis after percutaneous coronary intervention (PCI) in coronary heart disease in Hai’an and construction of risk prediction model. J Integr Tradit Chin West Med Cardiocerebral Vasc Dis. (2024) 22(03):542–7. 10.12102/j.issn.1672-1349.2024.03.027

[17] Bai X-L Jiang X Pang J Yang Z-Z Wei Q. To construct and validate a risk prediction model for restenosis or recurrent myocardial infarction after percutaneous coronary intervention (PCI). Mil Nurs. (2024) 41(02):11–5. 10.3969/j.issn.2097-1826.2024.02.003

[18] Wu S-B Wang W-Z Wu C. To construct a risk prediction model for in-stent restenosis (ISR) in percutaneous coronary intervention (PCI) patients based on glucose and lipid metabolism. J Integr Tradit Chin West Med Cardiocerebral Vasc Dis. (2023) 21(08):1459–64. 10.12102/j.issn.1672-1349.2023.08.019

[19] Jia L-L Zhang B. Establishment and validation of a nomogram model for predicting in-stent restenosis after percutaneous coronary intervention (PCI) in hypertensive patients with coronary heart disease. Chin J Arterioscler. (2023) 31(02):148–56. 10.3969/j.issn.1007-3949.2023.02.008

[20] Scafa-udriste A Itu L Puiu A Stoian A Moldovan H Popa-Fotea N-M. In-stent restenosis in acute coronary syndrome—a classic and a machine learning approach. Front Cardiovasc Med. (2023) 10:1270986. 10.3389/fcvm.2023.1270986 38204799 PMC10777838

[21] Guidener U Kessler T Von Scheidt M Scheidt M-V Hawe J-S Gerhard B Machine learning identifies new predictors on restenosis risk after coronary artery stenting in 10,004 patients with surveillance angiography. J Clin Med. (2023) 12(8):2941. 10.3390/jcm12082941 37109283 PMC10142067

[22] Coughan J-J Aytekin A Lahu S Scalamogna M Wiebe J Pinieck S Derivation and validation of the ISAR score to predict the risk of repeat percutaneous coronary intervention for recurrent drug-eluting stent restenosis. EuroIntervention. (2023) 18(16):e1328–38. 10.4244/EIJ-D-22-00860 36785947 PMC10068863

[23] Jiang Z Tian L Liu W Song B Xue C Li T-Z Random forest vs. logistic regression: predicting angiographic in-stent restenosis after second-generation drug-eluting stent implantation. PLoS One. (2022) 17(5):e268757. 10.1371/journal.pone.0268757 PMC9126385 35604911

[24] Luo Y Tan N Zhao J Li Y-H. A nomogram for predicting in-stent restenosis risk in patients undergoing percutaneous coronary intervention: a population-based analysis. Int J Gen Med. (2022) 15:2451–61. 10.2147/IJGM.S357250 35264881 PMC8901259

[25] Lin X-L Li Q-Y Zhao D-H Liu J-H Fan Q. Serum glycated albumin is associated with in-stent restenosis in patients with acute coronary syndrome after percutaneous coronary intervention with drug-eluting stents: an observational study. Front Cardiovasc Med. (2022) 9:943185. 10.3389/fcvm.2022.943185 36237913 PMC9551162

[26] Yi M Tang W-H Xu S Ke X Liu Q. Investigation into the risk factors related to in-stent restenosis in elderly patients with coronary heart disease and type 2 diabetes within 2 years after the first drug-eluting stent implantation. Front Cardiovasc Med. (2022) 9:837330. 10.3389/fcvm.2022.837330 35669469 PMC9163371

[27] Wang J Yang Y-C Zhang L He P-Y Mu H. Predictors of stent restenosis in Han and Uygur patients with coronary heart disease after PCI in the Xinjiang region. Cardiol Res Pract. (2022) 2022:7845108. 10.1155/2022/7845108 35958295 PMC9357810

[28] He W Xu C Wang X Lei J-Y Qiu Q-F Hu Y-Y Development and validation of a risk prediction nomogram for in-stent restenosis in patients undergoing percutaneous coronary intervention. BMC Cardiovasc Disord. (2021) 21(1):435. 10.1186/s12872-021-02255-4 34521385 PMC8442286

[29] Gai M-T Zhu B Chen X-C Liu F Xie X Gao X-M A prediction model based on platelet parameters, lipid levels, and angiographic characteristics to predict in-stent restenosis in coronary artery disease patients implanted with drug-eluting stents. Lipids Health Dis. (2021) 20(1):118. 10.1186/s12944-021-01553-2 34587955 PMC8480001

[30] Zhao J Wang X Wang H-Y Zhao Y Fu X-H. Occurrence and predictive factors of restenosis in coronary heart disease patients underwent sirolimus-eluting stent implantation. Ir J Med Sci. (2020) 189(3):907–15. 10.1007/s11845-020-02176-9 31989420

[31] Sampedro-Gomez J Dorado-Diza P-I Vicente-Palacios V Sánchez-Puente A Jiménez-Navarro M Roman J-A-S Machine learning to predict stent restenosis based on daily demographic, clinical, and angiographic characteristics. Can J Cardiol. (2020) 36(10):1624–32. 10.1016/j.cjca.2020.01.027 32311312

[32] Kang S-J Cho Y-R Park G-M Ahn J-M Han S-B Lee J-Y Predictors for functionally significant in-stent restenosis: an integrated analysis using coronary angiography, IVUS, and myocardial perfusion imaging. JACC Cardiovasc Imaging. (2013) 6(11):1183–90. 10.1016/j.jcmg.2013.09.006 24229771

[33] De-Jong Y Ramspek C-L Zoccali C Jager K-J Dekker F-W Van-Diepen M. Appraising prediction research: a guide and meta-review on bias and applicability assessment using the prediction model risk of bias ASsessment tool (PROBAST). Nephrology (Carlton). (2021) 26(12):939–47. 10.1111/nep.13913 34138495 PMC9291738

[34] Wang L-Y Zhang L-M Li J-N Du T-H. Risk factors of in-stent restenosis after percutaneous coronary intervention in patients with coronary heart disease: a meta-analysis. Nurs Res. (2021) 35(10):1711–9. 10.12102/j.issn.1009-6493.2021.10.003

[35] Shigematsu S Takahashi N Hara M Hara M Yoshimatsu H Saikawa T. Increased incidence of coronary in-stent restenosis in type 2 diabetic patients is related to elevated serum malondialdehyde-modified low-density lipoprotein. Circ J. (2007) 71(11):1697–702. 10.1253/circj.71.1697 17965487

[36] Chai D Yang X Wang A Lu S Dai Y-X Zhou J. Usefulness of platelet distribution width and fibrinogen in predicting in-stent restenosis with stable angina and type 2 patients with diabetes Mellitus. Front Cardiovasc Med. (2022) 9:710804. 10.3389/fcvm.2022.710804 35387442 PMC8977890

[37] Zheng J-F Guo T-T Wang Y Hu X-Y Chang Y Tian Y Short-term outcomes and prognostic risk factors of late drug-eluting stent restenosis patients undergoing repeated percutaneous coronary intervention. Chin Circ J. (2020) 35(4):349–54. 10.3969/j.issn1000-3614.2020.04.006

[38] Cassese S Byrne R-A Tada T Pinieck S Joner M Ibrahim T Incidence and predictors of restenosis after coronary stenting in 10 004 patients with surveillance angiography. Heart. (2014) 100(2):153–9. 10.1136/heartjnl-2013-304933 24270744

[39] Ullrich H Olschewski M Münzel T Gori T. Coronary in-stent restenosis: predictors and treatment. Dtsch Arztebl Int. (2021) 118(38):637–44. 10.3238/arztebl.m2021.0254 34379053 PMC8715314

[40] Elezi S Dibra A Mehilli J Pache J Wessely R Schömig A Vessel size and outcome after coronary drug-eluting stent placement: results from a large cohort of patients treated with sirolimus- or paclitaxel-eluting stents. J Am Coll Cardiol. (2006) 48(7):1304–9. 10.1016/j.jacc.2006.05.068 17010786

[41] Gan T Guan H Li P Huang X Li Y Zhang R Risk prediction models for cardiovascular events in hemodialysis patients: a systematic review. Semin Dial. (2024) 37(2):101–9. 10.1111/sdi.13181 37743062

[42] Akazawa M Hashimoto K Katsuhiko N Kaname N. Machine learning approach for the prediction of postpartum hemorrhage in vaginal birth. Sci Rep. (2021) 1:22620. 10.1038/s41598-021-02198-y 34799687 PMC8604915

[43] Venkatesh K-K Strauss R-A Grotegut C-A Heine R-P Chescheir N-C Stringer J-S-A Machine learning and statistical models to predict postpartum hemorrhage. Obstet Gynecol. (2020) 4:935–44. 10.1097/AOG.0000000000003759 32168227 PMC7236480

